# Auditory rhetoric in music: a perceptual framework for music-induced prosocial behavior

**DOI:** 10.3389/fpsyg.2026.1839326

**Published:** 2026-05-26

**Authors:** Jun-Yi Chen, Ting-Ting Liu, Xin-Wen Yao

**Affiliations:** 1 Fuzhou University of International Studies and Trade, Fuzhou, China; 2 Research Center for the Inheritance and Innovation of Handicraft Culture of Humanities and Social Science Base of Fujian Province Universities, Fuzhou, China; 3 Chengdu University of Technology, Chengdu, China

**Keywords:** auditory rhetoric, empathy, music cognition, music perception, perceptual alignment, prosocial behavior, relational orientation, social bonding

## Abstract

Music has been widely associated with prosocial behavior, including empathy, cooperation, and social bonding. Existing research has often explained these effects through specific mechanisms such as emotional responses, interpersonal synchrony, and neurophysiological processes. This study offers an alternative perspective by conceptualizing auditory rhetoric as a perceptual framework through which music organizes experience in ways relevant to prosociality. Drawing on research in music perception, attention, and emotion, this paper argues that music can be understood as a structured perceptual system that shapes how individuals attend to, interpret, and experience their environment. Within this framework, auditory rhetoric operates through three interrelated dimensions: structural organization, affective shaping, and attentional guidance. These dimensions help account for forms of perceptual alignment, including temporal, affective, and attentional alignment across individuals. Such alignment can support a shift toward relational orientation, characterized by a shared experiential frame and a reduced distinction between self and others. From this perspective, empathy, cooperation, social bonding, and prosocial decision-making become more likely when music helps establish these interpersonal conditions. By integrating perceptual, affective, and social processes within a more connected explanatory structure, this study provides an empirically informed framework for understanding how music contributes to prosocial behavior. This perspective contributes to the psychological understanding of music by framing prosociality in relation to perceptual organization rather than isolated mechanisms. It also highlights the importance of cultural context and musical features in shaping these processes, suggesting directions for future research in music psychology, social cognition, and cross-cultural studies.

## 1 Introduction

Music has increasingly been examined in relation to prosocial behavior, particularly in domains such as social bonding, cooperation, and trust. A growing body of empirical research indicates that both music listening and active musical engagement can enhance interpersonal connection across diverse contexts. Group musical activities, such as singing, have been associated with increased connectivity and elevated pain thresholds, suggesting their role in facilitating social bonding (Weinstein et al., 2016). Similarly, synchrony during dance has been shown to raise pain thresholds and encourage social bonding (Tarr et al., 2015), while coordinated movement and shared rhythmic engagement have been linked to increased cooperation (Lang et al., 2017). Experimental and intervention-based studies further demonstrate that music can promote prosocial tendencies, including empathy and cooperative decision-making, particularly when emotional engagement is involved (McDonald et al., 2022; Martí-Vilar et al., 2023; Ma et al., 2024).

Current research has largely approached music-induced prosociality through emotional, cognitive, and neurophysiological processes, with particular attention to empathy, interpersonal synchrony, and social bonding (McDonald et al., 2022; Juslin, 2021; Stupacher et al., 2017; Tarr et al., 2014). These approaches consistently highlight the roles of affective engagement, coordinated action, and neurohormonal responses—such as endorphin and oxytocin release—in shaping prosocial outcomes (Dunbar et al., 2012; Bowling et al., 2022; Chu and Tsai, 2026). Building on this line of research, the present study conceptualizes auditory rhetoric as a perceptual mechanism through which music structures experience in ways that support relational orientation. Specifically, auditory rhetoric refers to the organization of sound that guides attention, shapes affective experience, and aligns listeners within a shared experiential frame. From this perspective, music-induced prosociality may be more clearly understood when examined through perceptual alignment and relational orientation, offering a framework for explaining how musical organization contributes to social behavior.

Within this framework, auditory rhetoric is analytically understood through three interrelated dimensions: structural organization, affective shaping, and attentional guidance. The structural dimension refers to the temporal and rhythmic organization of sound, which enables alignment across listeners through patterned repetition and synchronization. The affective dimension concerns the ways in which musical features shape emotional experience, facilitating shared affective states among individuals. The attentional dimension involves the capacity of sound to guide focus, salience, and perceptual orientation within a shared auditory environment.

**Figure 1:**
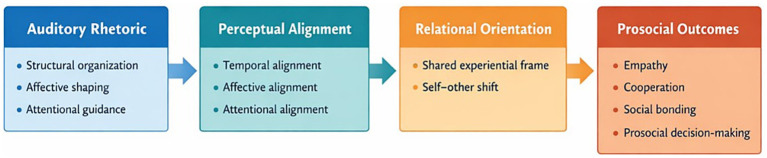
Auditory rhetoric as a perceptual pathway to prosocial behavior.

These dimensions collectively describe how music structures perceptual experience in ways that extend beyond individual processing and support relational orientation. Through the alignment of temporal patterns, affective responses, and attentional focus, auditory rhetoric helps explain how listeners may shift from self-oriented perception toward a shared experiential frame. In this sense, perceptual alignment provides a basis for interpersonal connection and coordinated action, and offers a way of understanding how music may give rise to prosocial tendencies. To illustrate the proposed framework, Figure 1 presents the conceptual model of auditory rhetoric as a perceptual pathway leading to prosocial behavior.

## 2 Auditory rhetoric in music

Music can be understood not merely as a sequence of sounds but as a structured perceptual system that organizes auditory information into meaningful patterns. Research in cognitive science and auditory neuroscience demonstrates that music is processed through hierarchical structures involving rhythm, pitch, and harmony, which are integrated into coherent perceptual representations (Dowling, 2012; Koelsch and Siebel, 2005; Mehr, 2025). These structures are not passively received but actively interpreted, as listeners rely on learned perceptual frameworks shaped by cultural exposure and prior experience to anticipate and organize musical events (Dowling, 2012; Pearce, 2025). In this sense, music functions as an organized perceptual environment rather than a simple acoustic stimulus.

Within this structured system, music plays a crucial role in guiding attention and organizing perceptual experience. Auditory attention is not evenly distributed but is shaped by the structural features of sound, allowing listeners to selectively focus on salient elements within complex auditory scenes (Carlyon and Cusack, 2005; Bürgel et al., 2021). Rhythmic and temporal regularities further entrain attention over time, facilitating coordinated perceptual processing and enhancing the integration of multiple auditory streams (Brannick and LaCroix, 2025; Rosário et al., 2020). Neurophysiological evidence suggests that such attentional organization reflects dynamic interactions between neural processes and musical structure, enabling listeners to experience music as a coherent and unified whole (Koelsch and Skeie, 2020; Harding et al., 2025).

Music also shapes emotional experience through its structural and acoustic properties. Features such as tempo, dynamics, and harmony contribute to the emergence of emotional responses by creating patterns of tension, anticipation, and resolution (Arjmand et al., 2017; Greer et al., 2019). These responses are not fixed but arise from the interaction between musical structure and individual listener characteristics, resulting in complex and often blended emotional experiences (Xu et al., 2021; Zhang et al., 2019). Rather than simply eliciting discrete emotions, music organizes affective experience in ways that are dynamically constructed and context-dependent (Alaminos Fernández, 2014).

From a rhetorical perspective, perception and experience are not neutral processes but are shaped by the organization of sensory and symbolic elements. Rhetorical theory has long emphasized that meaning is produced through the structuring of perception, where sensory engagement plays a central role in shaping understanding and response (Poole, 2020; Hawhee, 2011). In this view, rhetoric extends beyond persuasion to include the organization of experience itself, often through embodied and multimodal processes that integrate sensory input and cognitive interpretation (Fountain, 2014; Aczél, 2023).

Building on these perspectives, auditory rhetoric can be conceptualized as the organization of sound that structures perception, guides attention, and shapes affective experience. Rather than treating music as a passive medium or a mere trigger of internal states, this approach positions musical sound as an active organizer of perceptual experience. In doing so, it provides a conceptual foundation for understanding how music can align individuals within a shared experiential framework, thereby supporting the emergence of relational orientation and prosocial behavior.

## 3 Auditory rhetoric and relational orientation

### 3.1 Temporal alignment and interpersonal synchrony

A substantial body of research indicates that musical synchrony plays a central role in shaping social bonding and cooperative behavior. Interpersonal synchrony, defined as the temporal alignment of actions or movements between individuals, has been shown to enhance perceived social closeness and facilitate coordinated interaction (Tarr et al., 2016; Yu et al., 2025; Bamford et al., 2023). Musical contexts provide a particularly effective medium for such alignment, as rhythmic structures guide individuals toward shared timing and coordinated behavior. Empirical studies demonstrate that synchronized movement during music, such as dance, can increase pain thresholds and social closeness, suggesting a link between synchrony, physiological response, and bonding (Tarr et al., 2016; Dunbar et al., 2012). In addition, synchrony has been shown to signal group cohesion and influence intergroup perception, reinforcing social connectedness (Good et al., 2017; Lee et al., 2020). Importantly, the effects of synchrony extend across developmental stages. Evidence indicates that even infants use interpersonal synchrony as a cue for prosocial behavior, directing helping behavior toward individuals with whom they have previously been synchronized (Cirelli et al., 2014). Musical synchrony has also been associated with interbrain synchronization and coordinated physiological responses, further supporting its role in facilitating social alignment (Ding et al., 2025; Kang et al., 2023; Lavezzo et al., 2025). Together, these findings suggest that temporal alignment functions as a foundational mechanism through which music supports relational orientation.

### 3.2 Affective alignment and shared emotional experience

Music also shapes prosocial behavior through shared emotional experience. A growing body of research demonstrates that music-induced emotions influence empathy, compassion, and prosocial decision-making (Wu et al., 2024; McDonald et al., 2022). Emotional engagement with music has been shown to increase helping behavior and enhance sensitivity to others’ emotional states, particularly when individuals experience similar affective responses (Wu et al., 2024). Moreover, shared musical experiences amplify emotional responses and strengthen interpersonal connection. This relational effect may be further shaped by individual empathic capacity, as higher empathy has been associated with stronger social bonding when people move together with music (Stupacher et al., 2022). Studies indicate that collective engagement with music enhances pleasure and emotional intensity, which in turn contributes to social bonding and prosocial tendencies (Curzel et al., 2024; Rabinowitch and Gill, 2021). Emotional convergence, in which individuals experience aligned affective states, plays a key role in reducing perceived social distance and fostering group cohesion (Yu et al., 2025; Ding et al., 2025). Related evidence also suggests that empathic accuracy and affect sharing are linked not only in responses to people, but also in responses to music, indicating that music-related affective engagement may be tied to broader interpersonal capacities for shared emotional understanding (Tabak et al., 2022). These findings suggest that music-induced affect operates not only as an internal psychological process but also as a socially distributed mechanism linking individuals through shared emotional experience.

### 3.3 Attentional alignment and shared perceptual frame

A third dimension through which music facilitates prosociality is the alignment of attention and perception. Musical structure provides a temporal and sensory framework that guides listeners’ focus, enabling coordinated perception within a shared environment. More direct evidence for this attentional dimension can be found in studies of joint and shared attention in musical settings. For example, research on piano duos shows that focus of attention shapes experiences of togetherness and enjoyment, with joint and mutual attention producing stronger interpersonal connection than self-directed attention (Bishop, 2025). Related work also suggests that joint attention can enhance social bonding even in minimal shared-attention contexts (Wolf et al., 2016), while shared musical activity has been associated with stronger relationship commitment and interpersonal coordination (Harwood and Wallace, 2022). Research on collective music listening shows that rhythmic and sensory cues enhance movement coordination and shared engagement, particularly under conditions of strong groove and social interaction (Dotov et al., 2021; Stupacher et al., 2020). Visual and auditory cues in musical interaction further support shared attention, as individuals rely on both modalities to coordinate perception and action (Pentimalli and Gobo, 2023). In addition, shared auditory experiences can produce synchronized neural activity, reflecting alignment at the level of perception and cognition (Ding et al., 2025; Kang et al., 2023). Cultural familiarity and shared musical preferences also influence how individuals attend to and interpret musical stimuli, shaping collective perception and interpersonal connection (Stupacher et al., 2020; Bakagiannis and Tarrant, 2006). Through these processes, music establishes a shared perceptual frame that supports coordinated attention and collective experience.

### 3.4 From alignment to relational orientation

Alignment can be understood as more than a perceptual effect. When musical experience is shared, coordination in timing, feeling, and attention may begin to develop into a more relational state marked by social closeness, connectedness, and a reduced sense of interpersonal distance. Several recent studies point in this direction. For example, music co-audition has been shown to enhance emotional alignment and group cohesion at both behavioral and neural levels (Ding et al., 2025). Related research also suggests that music-induced emotions, particularly when accompanied by mental imagery, are associated with stronger feelings of social connectedness (Xu et al., 2026), while joint music listening can enhance interpersonal affective and neural synchrony (Curzel et al., 2026). Studies of joint attention in musical and shared-attention settings likewise suggest that attending together can strengthen togetherness and social bonding, indicating that attentional alignment may also contribute to the emergence of relational states (Bishop, 2025; Wolf et al., 2016). Likewise, studies of synchronized musical engagement indicate that moving together and sharing musical enjoyment can reinforce social closeness (Stupacher et al., 2020; Yu et al., 2025). Rather than remaining separate perceptual effects, these forms of alignment appear to create the conditions for a more shared experiential orientation. Temporal, affective, and attentional alignment together describe complementary processes through which music structures social experience. Rather than operating independently, these dimensions interact to produce a shift from individual perception toward a relational mode of experience. This shift can be conceptualized as relational orientation, in which individuals experience themselves as aligned with others within a shared perceptual and social frame. Through synchronized timing, shared emotional states, and coordinated attention, music facilitates perceptual alignment that supports interpersonal connection and collective engagement (Tarr et al., 2016; Wu et al., 2024; Dotov et al., 2021). Within this framework, prosocial behavior can be understood as emerging from relational orientation rather than isolated psychological mechanisms. Music organizes experience in ways that align individuals within a shared perceptual and social space, providing the conditions under which cooperation, empathy, and social bonding become more likely. To summarize the relationships between auditory rhetoric dimensions, alignment processes, and their associated social effects, Table 1 provides an overview of the corresponding pathways and representative empirical evidence.

**Table 1:** Auditory rhetoric dimensions, alignment processes, and prosocial outcomes.

Dimension	Perceptual function	Alignment process	Relational effect	Prosocial outcome	Key references
Structural	Temporal organization of sound	Interpersonal synchrony	Coordination and shared timing	Social bonding	Tarr et al. (2016), Yu et al. (2025), and Cirelli et al. (2014)
Affective	Emotional shaping and affective modulation	Shared emotional experience	Emotional convergence	Empathy and compassion	McDonald et al. (2022), Wu et al. (2024), and Colverson et al. (2021)
Attentional	Guidance of perceptual focus	Shared attention	Joint perceptual orientation	Cooperation and collective action	Dotov et al. (2021), Ding et al. (2025), and Stupacher et al. (2020)

## 4 Relational orientation and prosocial functions

### 4.1 Empathy and affective connection

A growing body of research demonstrates that music plays a significant role in enhancing empathy and compassion, which are central to prosocial behavior. Emotional music has been shown to increase empathy and compassionate responses, particularly in socially relevant contexts (McDonald et al., 2022). Similarly, empirical studies indicate that musical engagement is associated with higher levels of empathic decision-making and emotional sensitivity (Colverson et al., 2021; Hua et al., 2025). Participation in musical activities, such as ensemble performance, further strengthens empathy by requiring individuals to coordinate emotionally and cognitively with others (Cho and Han, 2022). In addition, long-term musical training has been linked to increased empathy and prosocial tendencies, suggesting that music may shape social cognition over time (Morand-Grondin et al., 2026).

### 4.2 Prosocial behavior and cooperative action

Music also directly influences prosocial behavior, including helping, sharing, and charitable actions. Experimental evidence shows that exposure to music with prosocial lyrics increases helping behavior and generosity, likely by activating prosocial thoughts and attentional biases toward others’ needs (Greitemeyer, 2009; Ruth, 2017). More recent studies further demonstrate that music can enhance charitable decision-making and intertemporal generosity, indicating its influence on prosocial decision processes (Hong et al., 2023; Hong et al., 2025). In developmental contexts, musical activities have been found to promote prosocial behaviors among children, highlighting the role of music in early social development (Ma et al., 2024). Neurocognitive evidence also suggests that music can influence implicit cognition and prosocial responses, further supporting its behavioral impact (Xu et al., 2025).

### 4.3 Social bonding and collective identity

Beyond individual behavior, music plays a crucial role in shaping social bonding and group identity. Group musical activities, such as singing, have been shown to increase social closeness and collective cohesion, often accompanied by physiological changes such as elevated pain thresholds (Weinstein et al., 2016). Theoretical and empirical work suggests that music facilitates “self–other merging” through synchrony and shared experience, thereby strengthening interpersonal bonds (Tarr et al., 2014). Similarly, interpersonal synchrony in musical contexts promotes social bonding through coordinated activity and shared physiological responses (Yu et al., 2025). At a broader level, music also contributes to the construction of group identity. Shared musical preferences have been shown to signal value similarity, thereby increasing social attraction and group cohesion (Boer et al., 2011). Collective musical participation, such as group singing, further enhances feelings of belonging and community, reinforcing social identity through shared experience (Camlin et al., 2020).

### 4.4 Prosocial decision-making and moral evaluation

Music additionally influences prosocial decision-making by shaping emotional states and cognitive evaluations. Emotional music has been shown to enhance prosocial decisions, particularly by increasing empathy and sensitivity to others’ suffering (Wu et al., 2024). Studies also indicate that background music can affect decision-making processes, including risk-taking and temporal preferences, suggesting that music shapes evaluative judgment in social contexts (Israel et al., 2019; Israel et al., 2022). Furthermore, music can influence moral evaluation, as individuals are more likely to judge harm as wrong when musical cues enhance emotional engagement (Agrawal et al., 2023). Research on cooperative tasks also indicates that music can facilitate coordination and influence decision outcomes, highlighting its role in shaping social behavior beyond immediate emotional responses (Liebman et al., 2018).

### 4.5 From prosocial outcomes to relational orientation

Prosocial outcomes in music do not need to be understood as separate end results. They become easier to explain when read in relation to the interpersonal states that shared musical experience helps bring about. This connection is also consistent with empirical research showing that social closeness, empathy, and related interpersonal capacities shape how individuals respond to others. Social closeness has been shown to influence decision-making in contexts involving prosocial orientation (Fu et al., 2025). Related work further indicates that interpersonal closeness enhances mutual empathy at both behavioral and neural levels (Lin et al., 2024), while empathy itself can give rise to more stable forms of social closeness over time (Saulin et al., 2024). Other studies likewise suggest that empathy, empathic accuracy, and related interpersonal capacities are positively associated with prosocial behavior, helping, and social responsiveness (Eckland et al., 2020; Gungordu et al., 2025; Marshall et al., 2020). From this perspective, empathy, cooperation, social bonding, and prosocial decision-making are not simply isolated outcomes, but manifestations of a more relational mode of experience. Within the present framework, music matters because it helps create the interpersonal conditions under which such prosocial tendencies become more likely to emerge.

## 5 Discussion

This study advances a perceptual account of music-induced prosociality by conceptualizing auditory rhetoric as a mechanism through which musical structure organizes experience and supports relational orientation. Across the preceding sections, evidence from cognitive, affective, and social domains demonstrates that music influences prosocial outcomes through processes such as interpersonal synchrony, shared emotional experience, and coordinated attention. By integrating these processes within a unified perceptual framework, this study offers a coherent explanation of how music facilitates prosocial tendencies.

A key contribution of this perspective lies in reframing music-induced prosociality not as the result of isolated mechanisms, but as an emergent property of perceptual alignment. Existing research has more often approached these effects through partial mechanisms than through a single integrated pathway. In music-related studies, prosocial effects are frequently examined through synchrony, emotional response, empathy, social bonding, or neural alignment, with each mechanism illuminating part of the process rather than the full sequence at once (Ding et al., 2025; Grimani et al., 2024; Pardo-Olmos et al., 2025; Tzanaki, 2022; Wu et al., 2024). From this perspective, the value of the present framework lies not in replacing those accounts, but in bringing them into a more connected explanatory structure. In contrast, the present framework highlights how temporal coordination, affective convergence, and attentional alignment are interconnected through the organization of auditory experience. Together, these dimensions support a shift from self-oriented perception toward a shared experiential frame, thereby enabling relational orientation and prosocial behavior.

This perspective also contributes to the broader psychological understanding of music by emphasizing the role of perception as a socially organizing process. Rather than viewing perception as an individual and passive mechanism, auditory rhetoric positions perceptual experience as dynamically structured and inherently relational. In this sense, music does not merely influence internal states such as emotion or cognition; it actively organizes how individuals attend to, interpret, and experience the social world. This helps to explain why music is particularly effective in fostering empathy, cooperation, and social bonding across diverse contexts.

Furthermore, the framework of auditory rhetoric provides a conceptual bridge across levels of analysis in music research. It connects low-level perceptual processes, such as temporal structuring and attentional guidance, with higher-level social outcomes, including group identity, interpersonal connection, and prosocial behavior. By linking these levels within a single explanatory model, the present study contributes to a more integrated understanding of music’s role in human social life.

At the same time, several limitations and directions for future research should be considered. First, although the present framework synthesizes findings across multiple domains, much of the existing evidence still supports specific segments of the process rather than a fully tested pathway. Some studies focus on synchrony and social bonding, others on music-induced emotion and prosocial decision-making, and others on empathy or neural alignment during shared musical experience (Ding et al., 2025; McDonald et al., 2022; Pardo-Olmos et al., 2025; Tzanaki et al., 2025; Wu et al., 2024). Future research may therefore examine more directly how perceptual alignment develops into relational orientation, and how this relational shift in turn predicts different forms of prosocial outcome across musical contexts.

Second, the role of cultural and contextual factors remains an important area for further exploration. Musical perception and emotional interpretation are shaped by cultural background, as individuals from different cultural contexts may interpret musical features and emotional cues in distinct ways (Li et al., 2025; Tang et al., 2025). These differences suggest that the operation of auditory rhetoric may vary across sociocultural environments, influencing how perceptual alignment and relational orientation are established.

Third, the extent to which different musical forms produce similar or divergent prosocial effects warrants further investigation. While musical genre itself may not directly determine prosocial outcomes, variations in musical features—such as emotional tone, arousal level, and lyrical content—have been shown to influence prosocial behavior and decision-making (Wu et al., 2024; Yu et al., 2019; Ma et al., 2023). This indicates that the prosocial potential of music may depend on how auditory structures are configured within specific musical contexts.

Finally, this perspective has implications for applied contexts. Understanding music as a form of auditory rhetoric suggests that musical interventions can be designed not only to evoke emotional responses but also to structure shared experience and promote social connection. Such an approach may be particularly relevant in educational, therapeutic, and community settings, where music can be used to foster cooperation, empathy, and collective engagement.

This figure illustrates the proposed framework of auditory rhetoric as a perceptual pathway to prosocial behavior. Auditory rhetoric, conceptualized as the structural, affective, and attentional organization of sound, shapes perceptual alignment across individuals. This alignment, including temporal, affective, and attentional synchronization, facilitates a shift toward relational orientation, characterized by a shared experiential frame and reduced self–other distinction. As a result, music supports a range of prosocial outcomes, including empathy, cooperation, social bonding, and prosocial decision-making.

This table summarizes the proposed relationships between auditory rhetoric dimensions, perceptual alignment processes, relational effects, and prosocial outcomes. The dimension column refers to the three core components of auditory rhetoric, including structural organization, affective shaping, and attentional guidance. The perceptual function column describes how musical sound is organized at the perceptual level. The alignment process column indicates the corresponding forms of alignment across individuals, including temporal, affective, and attentional alignment. The relational effect column reflects how these alignment processes contribute to relational orientation, such as shared experience and coordinated interaction. The prosocial outcome column presents the resulting prosocial tendencies associated with each dimension. Representative references are provided to illustrate empirical support for each pathway.

## Data Availability

The original contributions presented in the study are included in the article/supplementary material, further inquiries can be directed to the corresponding author/s.
